# AQP4-dependent enhancement of glymphatic function attenuates tau pathology and neurodegeneration in PS19 mice

**DOI:** 10.1186/s13024-026-00977-7

**Published:** 2026-07-18

**Authors:** Kaoru Yamada, Kazuhisa Ishida, Asami Sakamoto, Hitoshi Shimada, Masaki Watanabe, Masato Shimojo, Hironaka Igarashi, Takeshi Iwatsubo

**Affiliations:** 1 Department of Neuropathology, Graduate School of Medicine, The University of Tokyo, 7-3-1, Hongo, Bunkyo-ku, Tokyo, 113-0033 Japan; 2 Dementia Inclusion and Therapeutics, Graduate School of Medicine, The University of Tokyo Hospital, Tokyo, Japan; 3 Center for Integrated Human Brain Science, Brain Research Institute, Niigata University, Niigata, Japan; 4 Neuroscience Drug Discovery Unit, Takeda Pharmaceutical Co., Ltd. Osaka, Research, Japan; 5 National Institute of Neuroscience, National Center of Neurology and Psychiatry, Kodaira, Tokyo Japan

**Keywords:** Glymphatic system, Aquaporin-4, Tau pathology, Alzheimer’s disease, Cerebrospinal fluid dynamics

## Abstract

**Background:**

The glymphatic system facilitates cerebrospinal fluid–interstitial fluid exchange and contributes to the clearance of pathogenic proteins from the brain. Glymphatic dysfunction has been associated with Alzheimer’s disease and related tauopathies; however, whether impaired glymphatic transport causally drives tau accumulation and neurodegeneration, and whether its enhancement confers therapeutic benefit, remains unclear.

**Methods:**

Glymphatic water dynamics in PS19 tau transgenic mice were assessed using JJVCPE, a novel MRI-based approach for evaluating brain water exchange. The effect of pharmacological activation of aquaporin-4 (AQP4) with TGN-073 on glymphatic cerebrospinal fluid influx was examined in wild-type mice using dynamic contrast–enhanced MRI. Tau pathology, neurodegeneration, and cerebrospinal fluid tau levels were analyzed in PS19 mice following chronic TGN-073 treatment. AQP4-deficient PS19 mice were examined to determine target specificity.

**Results:**

PS19 mice exhibited significant impairment of glymphatic water exchange at early disease stages, which progressively worsened with ageing. Pharmacological activation of AQP4 with TGN-073 robustly enhanced glymphatic-related tracer influx, reduced tau accumulation, neuronal loss, and gliosis, and was accompanied by increased cerebrospinal fluid tau levels. TGN-073 also restored perivascular AQP4 enrichment without significantly altering overall AQP4 abundance. Importantly, these beneficial effects were abolished in AQP4-deficient PS19 mice, demonstrating that both glymphatic enhancement and suppression of tau pathology and neurodegeneration are AQP4-dependent.

**Conclusions:**

Our findings support a mechanistic contribution of impaired glymphatic function to tau accumulation and neuronal vulnerability in tauopathy. Pharmacological activation of AQP4 enhances glymphatic function, restores perivascular AQP4 organization, and ameliorates tau pathology, neurodegeneration, and gliosis. These findings identify AQP4-mediated glymphatic modulation as a disease-relevant and therapeutically tractable pathway for tau-related neurodegenerative disorders.

**Supplementary Information:**

The online version contains supplementary material available at 10.1186/s13024-026-00977-7.

## Background

The glymphatic system, first described over a decade ago, facilitates the clearance of interstitial solutes from the brain by driving CSF along perivascular spaces and promoting exchange with interstitial fluid (ISF) [1]. This process critically depends on aquaporin-4 (AQP4), a water channel highly expressed on astrocytic endfeet surrounding cerebral vasculature, which facilitates efficient water flux necessary for glymphatic function [2]. Since its initial proposal, accumulating evidence from animal and human studies has supported the existence of glymphatic flow and its relevance to brain homeostasis [3, 4]. In humans, glymphatic dysfunction has been implicated in Alzheimer’s disease (AD) [5, 6]. Notably, reductions in glymphatic function not only correlate with disease severity but can also predict subsequent amyloid-β (Aβ) accumulation [7], suggesting that impaired glymphatic clearance may actively contribute to AD pathogenesis. Reduced glymphatic flow is thought to impair clearance of extracellular Aβ [8], and accumulating Aβ in turn may further compromise glymphatic function [9, 10], establishing a vicious cycle that accelerates disease progression.

While interest in the glymphatic system continues to grow, it remains unclear whether pharmacological enhancement of glymphatic flow is a feasible and effective therapeutic strategy—particularly for tauopathies, where tau accumulation is a critical driver of neuronal loss and cognitive decline. Because glymphatic transport occurs primarily in the extracellular space, it was long assumed to affect only extracellular solutes such as Aβ [11, 12], leaving its potential influence on intracellular tau pathology uncertain. However, recent studies, including our own, have challenged this view by suggesting that glymphatic dysfunction may alter the extracellular tau pool available for trans-synaptic propagation, thereby indirectly shaping intracellular tau accumulation [13–15]. These findings raise the possibility that impaired glymphatic flow not only results from but also contributes to tau pathology, pointing toward a bidirectional relationship that has not been fully characterized.

To address these unresolved questions, we first examined whether progressive tau accumulation impairs glymphatic flow by visualizing water dynamics in PS19 tauopathy model mice using JJ vicinal coupling proton exchange (JJVCPE) MRI with H_2_^17^O [16]. We also evaluated age-related glymphatic changes in wild-type mice to distinguish tau-specific effects from normal aging. We further investigated whether pharmacologically enhancing glymphatic function using TGN-073—a blood–brain barrier–permeable small-molecule AQP4 enhancer—could mitigate tau pathology and neurodegeneration in PS19 mice, and validated target specificity in AQP4-deficient mice.

## Methods

### Mouse models and pharmacological treatment

For ^17^O-labeled water MRI (JJVCPE), male PS19 mice (B6;C3-Tg(Prnp-MAPT*P301S)PS19Vle/J, stock number: 008169, The Jackson Laboratory) that had been backcrossed with C57BL/6J mice for more than ten generations were used, together with their male littermate controls. For DCE-MRI study, ten-to-twelve weeks old male C57BL/6J mice were used. For histological, and biochemical experiments, PS19 mice over-expressing P301S mutant tau (B6;C3-Tg(Prnp-MAPT*P301S)PS19Vle/J, stock number: 008169, The Jackson laboratory) [17] and wildtype mice on B6C3F1 background were used. In some experiments, PS19 mice were crossed with AQP4 knockout mice on a C57BL/6J background, to generate PS19/AQP4KO mice [11, 13]. Both male and female mice (sex-balanced across groups) were included for the analysis. All animals were maintained under specific pathogen-free conditions with a 12-h light/dark cycle and ad libitum access to food and water. For chronic treatment, TGN-073 was converted to its sodium salt form to enable dissolution in water and was administered to PS19 mice or PS19/AQP4 KO mice in drinking water (400 mg/kg/day) starting at 6 months of age for 3 months.

### JJVCPE study

Mice, breathing spontaneously and anesthetized with an intra-peritoneal administration of urethane (1.2 g/kg), were positioned on their backs in a custom-made Plexiglas stereotactic holder. Their head was fixed in position by ear and tooth bars. Rectal temperature was maintained at 37 ± 0.5˚C using a custom-designed temperature control system. Ten mice of each group accomplished completed the experiment. Normal saline (6.6 ml/kg) containing 40% of H_2_^17^O was administered as an intravenous bolus injection at the 75th phase (10 min after the first scan) for 30 s through PE50 tubing inserted into the right femoral vein. MRI experiments were performed on a 15 cm bore 7 T horizontal magnet (Magnex Scientific, Abingdon, UK) with a Varian Unity-INOVA-300 system (Agilent Inc., Palo Alto, CA, USA) equipped with an actively shielded gradient. A custom made eight elements birdcage coil, 30 mm of inner diameter, was used for RF transmission, and quadrature oval surface coil with 20 mm outer diameter was used for RF receive. Adiabatic double-spin echo-prepared rapid acquisition with refocused echoes (RARE) was utilized with the following parameter settings: single slice (2 mm thick), 128 × 128 matrix image of 20 × 20 mm field of view for every 8 s, TR of 2000 milliseconds, Echo Train of 32, TE for first echo of 8.8 milliseconds, echo spacing of 5 milliseconds, and effective TE of 84.8 milliseconds. Imaging slabs were set 6 mm caudal from the top of the cerebrum. A total of 500 phases (scan time 70 min) were obtained at 8-second intervals.

Images were analyzed by image processing software (MR vision; MRVision Co. Winchester, MA, USA). Average % intensities, which reflect relative influx of H_2_^17^O in three areas namely cortex, basal ganglia (BG), and third ventricle, were plotted against time. Intensities at the steady state of each area, expressed as % against the averaged intensity of identical pixel prior to administration of H_2_^17^O, were determined by fitting their time course by the function. I = I0(1-ae-bt).

### Surgical preparation for DCE-MRI

Mice were initially anesthetized with isoflurane (3–5%) in oxygen for induction. For imaging sessions, anesthesia was maintained by a single intraperitoneal injection of a combination of medetomidine (0.75 mg/kg), midazolam (4 mg/kg), and butorphanol (5 mg/kg). This injectable regimen (Me/Mi/Bu) is widely used for surgical anesthesia in mice. After administering this anesthetic mixture, each mouse was secured in a stereotaxic frame, and the skin over the posterior neck was shaved. A midline incision was made to expose the dorsal atlanto-occipital membrane, and the muscles overlying the cisterna magna were gently retracted.

A pulled glass micropipette (~ 0.016 mm outer diameter) was positioned into the cisterna magna for contrast agent infusion. The pipette was fixed in place (e.g. with surgical glue) for the duration of the experiment. Gadobutrol (Gadavist; 1.0 M) was used as the contrast agent. A volume of 20 µL gadobutrol was infused into the subarachnoid space of the cisterna magna at a constant rate (approximately 0.5 µL/min) using a syringe pump. This intracisternal injection technique (shaving the neck, exposing the dura, and infusing a Gd-tracer at fixed rate) follows established protocols. After the infusion, the pipette was left in place briefly to prevent backflow, and then the wound was closed. Fifteen minutes prior to administration of contrast agent, TGN-073 (100 mg/kg) was administered by i.p.injection.

### Image processing and analysis for DCE-MRI

The acquired image series were first corrected for rigid-body motion. All time-point images were coregistered to the pre-injection baseline volume using a six-parameter affine (rigid) transform in FSL’s FLIRT software. This step mitigates motion artifacts while preserving signal changes due to contrast. Signal intensities were then normalized to each animal’s baseline volume to compute % signal change. No spatial smoothing was applied beyond any system defaults.

Regions of interest (ROIs) were manually drawn on the cortex and basal ganglia of each hemisphere using FIJI (ImageJ) or MRIcroGL, guided by anatomical landmarks. The mean signal intensity within each ROI was extracted at each time point. For each animal, the signal time course in each ROI was expressed as percentage change from baseline (pre-contrast) to account for any B1 inhomogeneity or baseline differences. These ROI-wise %ΔSI curves were then used to quantify glymphatic influx. In particular, the slope of the signal increase (infusion rate) and the area under the curve were calculated for each ROI. Group differences were assessed by repeated-measures analysis of variance at corresponding time points.

### CSF collection and analysis

CSF was collected from the cisterna magna under stereotaxic visualization. Mice were anesthetized with an intraperitoneal injection of a combined anesthetic (medetomidine 0.75 mg/kg, midazolam 4.0 mg/kg, butorphanol 5.0 mg/kg) and placed in a stereotaxic frame with the head flexed to approximately 90°. After shaving the dorsal neck region, a midline skin incision was made and overlying neck muscles were sequentially separated to expose the dura mater over the cisterna magna. Hemostasis was achieved as needed to prevent blood contamination. A 30G needle connected to FEP tubing was positioned above the dura under a dissecting microscope. The roller pump was initiated and the dura was gently punctured at the center of the triangular cisterna magna. CSF, under positive pressure, flowed spontaneously into the tubing. Once CSF entered the tubing, the pump speed was reduced to 1 µl/min to stabilize collection. After obtaining the desired volume, the needle was slowly withdrawn. CSF samples were visually inspected at the time of collection, and samples showing visible blood contamination were excluded from analysis.

### Histochemistry/Immunohistochemistry

To examine the tau pathology, gliosis, neuronal cell layers and perivascular AQP4 in brains, fixed hemi-brains were embedded in paraffin blocks and sectioned coronally at 4 μm thickness for histochemical analysis. De-paraffinized brain sections were rehydrated, and subjected to antigen retrieval. Sections were blocked with blocking solution and stained with hematoxylin and immunostained using PHF-1 or MC-1 antibody (kind gifts from Dr. Peter Davies) or NeuN antibody (Sigma Aldrich, MAB377). The sections were also immunostained using anti-GFAP (Cell signaling, #3670) or anti-Iba1 antibodies (Fujifilm, 019-19741). For antigen retrieval, de-paraffinzed sections were treated with microwave (550 W, 10 min) in citrate buffer (pH 6.0) prior to immunostaining. To detect proteinase K resistant tau inclusion, sections were treated with 100 µg/mL proteinase K for 6 min at 37 °C prior to PHF-1 staining [18].

To detect perivascular AQP4, sections were blocked with blocking solution and incubated overnight at 4 °C with primary antibodies against Aquaporin-4 (Sigma-Aldrich, HPA014784) and Collagen IV (SouthernBiotech, 1340-01). After washing, sections were incubated with species-appropriate fluorescent secondary antibodies.

### Biochemistry

Cortices were homogenized with RAB buffer containing cOmplete™ protease inhibitor cocktails (Roche) and PhosSTOP™ (Roche) and ultracentrifuged at 4 °C [19]. Supernatants were saved as RAB soluble fractions and stored at -80 °C. RAB insoluble pellets were homogenized with RIPA buffer containing cOmplete™ protease inhibitor cocktails (Roche) and PhosSTOP™ (Roche) and ultracentrifuged at 4 °C. Supernatants were saved as RIPA soluble fractions and stored at -80 C. RIPA insoluble pellets were homogenized with 70% formic acid (FA) and ultracentrifuged at 4 °C. Supernatants were saved as FA soluble fractions and stored at -80 °C.

### Human specific tau ELISA

Concentrations of human tau in CSF or brain homogenates were quantified by sandwich ELISAs using Tau-5 as a coating antibody and biotinylated HT7 (Thermo Fisher Scientific, MN1000B) as a detection antibody as described previously [20]. ELISA assays were performed using different lots of standard curves across experimental batches; thus, absolute concentrations are not directly comparable between assays.

### Image analysis

Bright-field images of immunostained brain sections were acquired using BZ-X1000 microscope (Keyence) equipped with a Plan-Apochromat 10x/0.45 NA objective lens at room temperature. Images were captured as tiled fields to reconstruct whole coronal sections of the cortex and hippocampus and automatically stitched using the Keyence Analyzer software. Final images were exported as 32-bit RGB TIFF files. No gamma correction was applied. For PHF-1 or MC1–stained sections, which included hematoxylin counterstaining, color deconvolution (H& DAB) was performed in Fiji (NIH) to isolate the DAB channel. Thresholding was then applied to the DAB image to quantify PHF-1 or MC-1–positive areas. For immunostaining, multiple sections (approximately from bregma − 2.0 to − 3.5 mm) per animal were quantified and averaged to generate a single value per mouse. Neuronal cell layer thickness of CA3 neurons and dentate granule cells and pyramidal cell layers in piriform cortex was measured using a scale perpendicular to the layer in two coronal sections stained with NeuN antibody. Hippocampal volume was estimated from NeuN-stained coronal sections at bregma − 2.0, − 2.5, and − 3.0 mm using the following formula: Volume between two sections = (average of the two sectional areas) × (distance between the sections).

Fluorescence images of AQP4 and collagen IV double-immunostained brain sections were acquired using a BZ-X1000 fluorescence microscope (Keyence) equipped with a Plan-Apochromat 20× objective lens at room temperature. Images were collected as tiled fields and stitched using the Keyence Analyzer software. Images were acquired as single-plane images using an autofocus function. AQP4 antibody specificity was validated using AQP4 knockout brain tissue, which showed minimal residual signal under identical imaging and analysis conditions.

For quantification of perivascular AQP4, hippocampal regions and hippocampal overlaying cortex were manually outlined on each section and used as regions of interest (ROIs) for all subsequent analyses. To focus on parenchymal microvascular AQP4 localization, large vessels located within the hippocampal molecular layer were excluded from the ROI prior to analysis. Collagen IV-positive vascular basement membrane structures were identified using a fixed intensity threshold applied uniformly across all images. Binary vascular masks were generated and used to define vascular structures. Perivascular regions were defined by expanding Collagen IV-positive masks using morphological dilation with a fixed number of pixels. The resulting dilated Collagen IV-based ROIs were overlaid onto the corresponding AQP4 channel images, and mean fluorescence intensity of AQP4 within these regions was quantified. Background fluorescence was defined as the mean signal intensity measured in PS19/AQP4 knockout sections and was used as a reference for background correction across all images. Total hippocampal AQP4 intensity was measured from the entire ROI in the corresponding AQP4 images. Multiple sections per animal were analyzed, and values were averaged for each mouse prior to statistical analysis.

### Y-maze spontaneous alteration test

Spontaneous alternation behavior was assessed using a Y-maze apparatus (each arm 35 cm long, 5 cm wide, 10 cm high). Mice were placed at the end of one arm and allowed to freely explore all three arms for 5 min. The entire session was recorded from above. An arm entry was counted and scored when the mouse’s hindquarters were completely inside the arm. Alternation behavior was defined as successive entries into three different arms on overlapping triplet sets (e.g., ABC, BCA, or CAB). The percentage of spontaneous alternations was calculated as (number of alternations / [total arm entries − 2]) × 100. The maze was wiped with water between trials to remove odor cues. Mice with fewer than 10 arm entries were excluded due to insufficient exploration.

### Limb clasping test

Motor impairment was assessed using the limb clasping test as previously described [21]. Mice were suspended by the tail and scored based on hindlimb posture using a 0–4 scale: 0 = normal extension of both hindlimbs, 1 = one hindlimb partially retracted, 2 = both hindlimbs partially retracted, 3 = one hindlimb fully clasped, and 4 = both hindlimbs fully clasped toward the body.

### Nest-building test

Nest-building behavior was evaluated as an indicator of social and motivational function. Each mouse was individually housed in a single cage, and two 2.5 g pressed cotton nestlets (Ancare) were provided at the beginning of the dark cycle. Nests were scored 24 h later on a 5-point scale as previously described [22].

### Statistics

Sample sizes for the experiments were determined based on prior publications using PS19 mice. All data were presented as mean ± S.E.M., and measurements were taken from distinct samples. Statistical analysis was performed using GraphPad Prism 9 (Graph Pad software). The normality of the data was verified using a Shapiro-Wilk test. For comparisons between two groups, Welch’s t-test was applied to account for potential inequality of variances. For comparisons between three groups, One-way ANOVA with Tukey’s post hoc test was applied. The relationship between hippocampal volume and tau pathology (PHF-1–positive or MC1-positive area) was assessed using Pearson’s correlation coefficient. For DCE-MRI experiments, the time course of signal intensity in each brain region was analyzed by two-way factorial ANOVA with Tukey’s post-hoc test. For quantitative analyses, outliers were identified using Grubbs’ test and excluded only when the test indicated a statistically significant outlier (*p* < 0.05). For discrete behavioral scores, including limb clasping and nestlet scores, comparisons between groups were performed using the Mann-Whitney U test.

### Use of artificial intelligence tools

Portions of the manuscript text were polished and edited using OpenAI’s ChatGPT (GPT3.5) to check for grammar and improve clarity and readability.

## Results

### Impaired glymphatic-related water exchange in PS19 mice revealed by JJVCPE MRI

Glymphatic dysfunction has been extensively characterized in amyloid-based models of Alzheimer’s disease (AD), but its relationship to tau pathology remains less defined. Although impaired glymphatic transport has been reported in tauopathy models [15, 23, 24], it is unclear how glymphatic function evolves across different stages of tau accumulation or how aging independently influences this process. To address these gaps, we assessed glymphatic water dynamics in wild-type and PS19 mice, focusing on age-related changes and their association with tau pathology. PS19 mice overexpress human tau carrying the P301S mutation and begin to develop hippocampal tau accumulation around 6 months of age, which subsequently spreads to the entire cerebral cortex by 9 months, accompanied by neuronal loss [17].

**Fig. 1:**
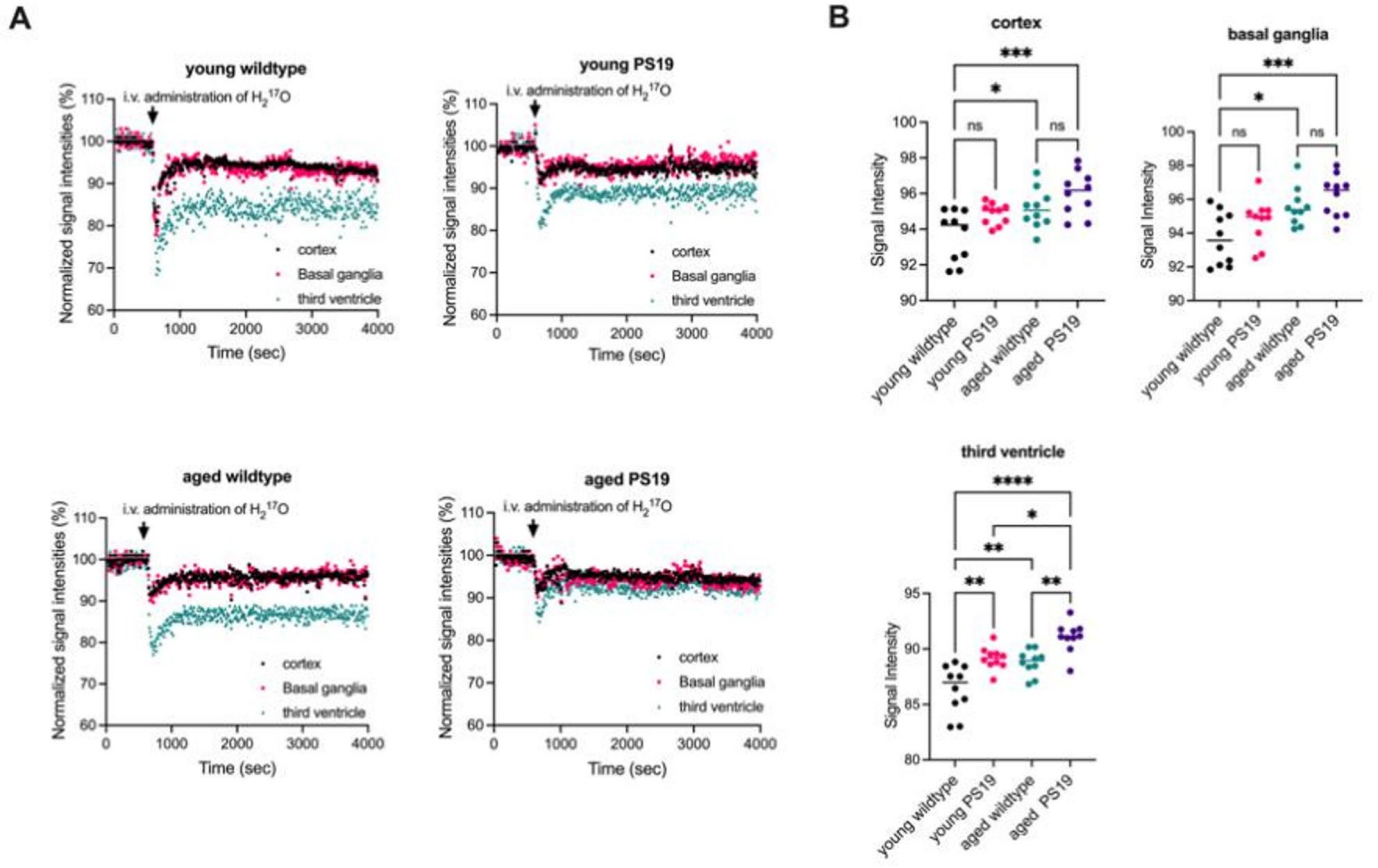
Age- and tau-dependent impairment of glymphatic water flow in PS19 mice revealed by JJVCPE MRI. (**A**) Representative time course of signal intensities in each ROI following intravenous H_2_^17^O administration in young (2–3 months) and aged (11–12 months) wild-type and PS19 mice. Signals in cortex, basal ganglia and 3rd ventricle are shown in black, magenta, cyan respectively. (**B**) Normalized signal intensity measured by JJVCPE MRI in cortex, basal ganglia, and third ventricle of young (2–3 months) and aged (11–12 months) wild-type and PS19 mice. Data are shown as mean ± SEM. Significant differences between groups are indicated (One-way ANOVA with Tukey’s post-hoc test. *p < 0.05, **p < 0.01, ***p < 0.001, ****p < 0.0001). *N* = 10 mice/group

To assess brain water dynamics, we used JJ vicinal coupling proton exchange (JJVCPE) MRI with intravenously injected H_2_^17^O. This method detects water movement based on ^17^O–^1^H proton exchange and enables tracer-free visualization of glymphatic-related water dynamics across vascular, interstitial, and CSF compartments, rather than bulk CSF flow [16]. Following intravenous injection of H_2_^17^O, most regions showed the characteristic JJVCPE signal pattern—a brief signal decrease followed by a gradual return to a steady-state plateau—reflecting rapid vascular delivery of H_2_^17^O and its subsequent equilibration within interstitial and CSF compartments (Fig. 1A). A higher steady-state signal indicates a reduced net increase in local H_2_^17^O concentration, consistent with impaired water exchange dynamics within the glymphatic system.

Previous JJVCPE studies demonstrated elevated third-ventricle steady-state signals in AQP4 knockout and APP/PS1 mice [16], indicating that this measure predominantly reflects AQP4-dependent glymphatic water exchange between parenchymal and CSF compartments rather than direct vascular entry.

Applying this approach to young (2–3 months) and aged (11–12 months) wild-type and PS19 mice (Fig. 1A), we found that cortical and basal ganglia signals were higher in aged mice but did not differ between genotypes, consistent with age-dependent slowing of water exchange (Fig. 1B). In contrast, third-ventricle signals increased with age and were significantly higher in PS19 mice at both ages (Fig. 1B). These findings confirm age-related glymphatic decline [25] and further indicate that PS19 mice exhibit early impairment in glymphatic water dynamics *before* overt tau accumulation. This early dysfunction mirrors observations in APP transgenic models, where glymphatic transport decreases before substantial Aβ deposition [10, 26]. Together, these results support a model in which early pathogenic tau species disrupt glymphatic flow, potentially creating a feed-forward loop that accelerates tau propagation and neurodegeneration.

### AQP4 activator TGN-073 enhances glymphatic influx in vivo

**Fig. 2:**
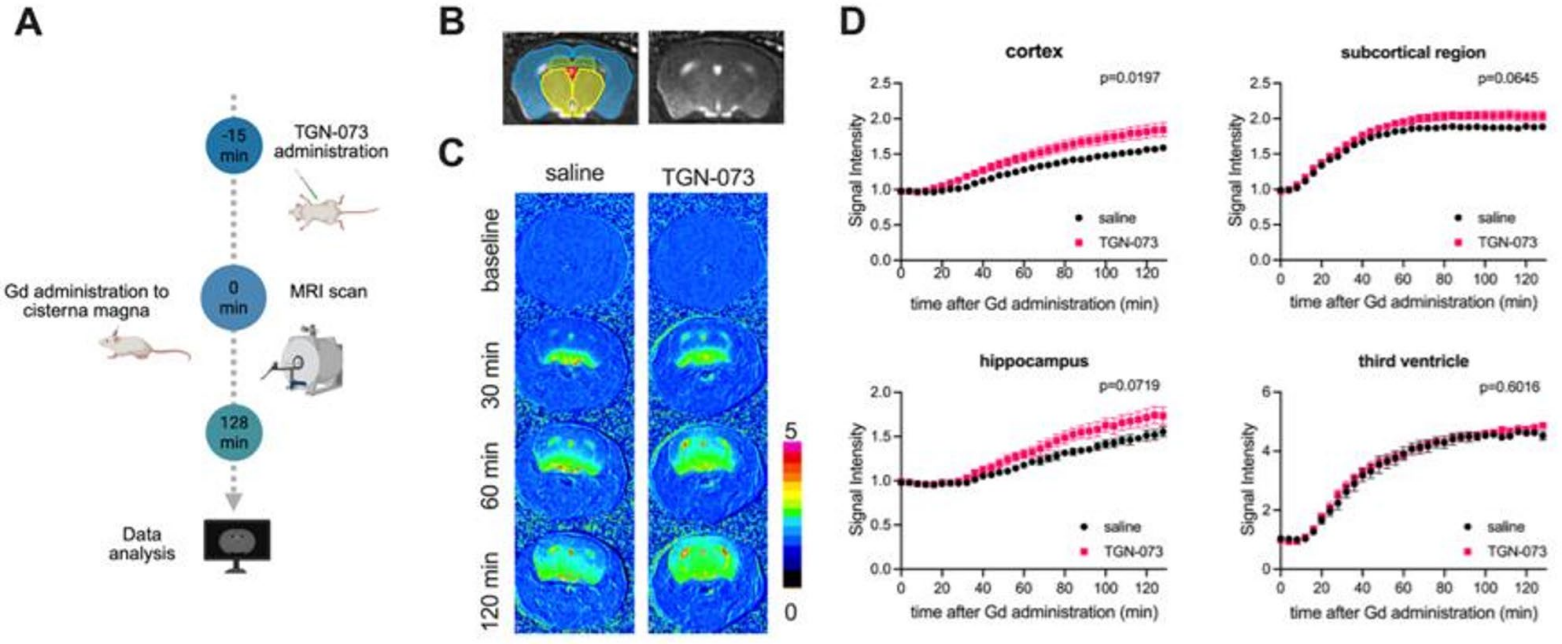
Pharmacological activation of AQP4 by TGN-073 enhances glymphatic influx in vivo. (**A**) Schematic illustration of the experimental setup for dynamic contrast-enhanced MRI (DCE-MRI) following intracisternal injection of gadoteridol (Gad) to visualize cerebrospinal fluid (CSF) influx into the brain parenchyma in wild-type mice pretreated with TGN-073 or saline. (**B**) Regions of interest (ROIs) in the MRI images are indicated as follows: the cortex (light blue), hippocampus (light green), third ventricle (red), and subcortical region (yellow). (**C**) Representative DCE-MRI images showing Gad distribution in the whole brain of wild-type mice pretreated with TGN-073 or saline, acquired at baseline, 30 min, 60 min, and 120 min. (**D**) Quantification of signal intensity over time in the hippocampus, cortex, and third ventricle. Two-way ANOVA with Tukey’s post-hoc test. Cortex: *p* = 0.0197; subcortical region: *p* = 0.0645; hippocampus: *p* = 0.0719; third ventricle: *p* = 0.6016. *N* = 5 mice/group

TGN-073 was previously identified as a small-molecule AQP4 enhancer using a Xenopus oocyte swelling assay [27]. To determine whether pharmacological enhancement of AQP4 facilitates glymphatic influx in vivo, we administered TGN-073 prior to dynamic contrast-enhanced MRI (DCE-MRI) following intracisternal injection of gadoteridol (Gad) (Fig. 2A). Unlike JJ vicinal coupling proton exchange (JJVCPE) MRI, which reflects overall water dynamics after intravenous H_2_^17^O administration, DCE-MRI allows direct visualization of CSF influx into the parenchyma.

In wild-type mice pretreated with TGN-073, Gad signal intensity increased more rapidly and broadly than in saline controls across multiple regions (Fig. 2B-D). The enhancement was most pronounced in the cortex, with similar but more modest trends in the hippocampus and subcortical areas. In contrast, third-ventricle signal curves were almost completely overlapping between groups, indicating that TGN-073 does not alter ventricular diffusion but instead selectively increases parenchymal influx. These findings demonstrate that pharmacological AQP4 activation augments glymphatic transport in vivo, especially in cortical regions relevant to tau pathology.

### Chronic TGN-073 treatment reduces tau pathology in PS19 mice

Building on prior findings that AQP4 deletion exacerbates tau pathology in PS19 mice [13], we next tested whether enhancing AQP4 function pharmacologically could mitigate tau accumulation. TGN-073 was administered chronically in drinking water (400 mg/kg/day) from 6 to 9 months of age, a regimen that was well tolerated without significant changes in body weight or behavior (supplemental Fig. 1A).

**Fig. 3:**
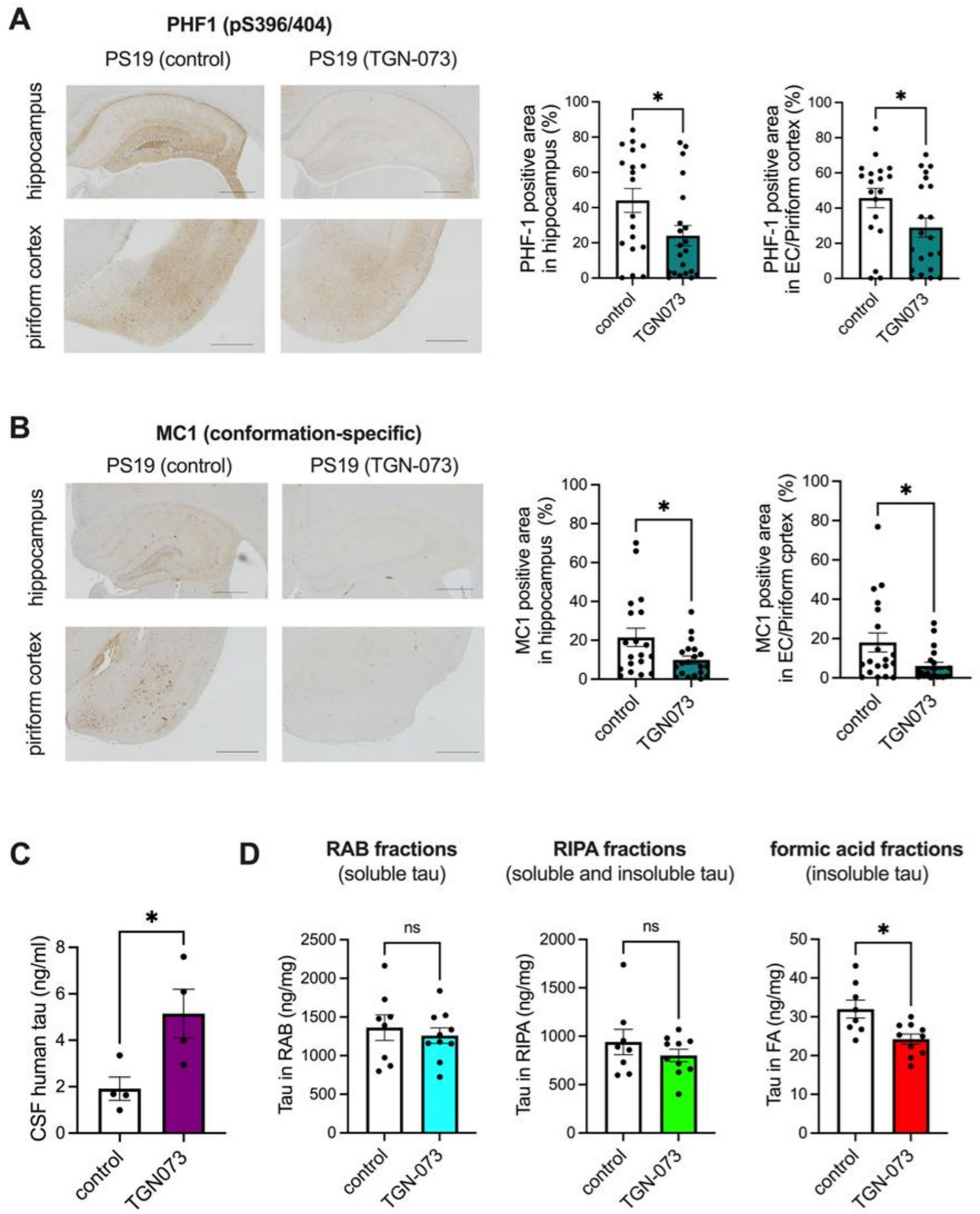
Chronic AQP4 activation by TGN-073 reduces tau pathology and insoluble tau accumulation in PS19 mice. (**A**) Representative images of the hippocampus and piriform cortex from 9-month-old PS19 mice treated with or without TGN-073 for 3 months, stained for PHF-1 with hematoxylin counterstaining. Scale bar, 500 μm. Quantification of PHF-1 positive area (%) in hippocampus or entorhinal (EC) /piriform cortex is shown. Unpaired two-tailed Welch’s t-test; *p < 0.05. *N* = 19–21 mice. Multiple sections across rostrocaudal levels (approximately bregma − 2.0 to − 3.5 mm) were analyzed in defined regions of interest (hippocampus, piriform cortex, and entorhinal cortex) and averaged per mouse. (**B**) Representative images of the hippocampus and piriform cortex from 9-month-old PS19 mice treated with or without TGN-073 for 3 months, stained for MC1. Scale bar, 500 μm. Quantification of MC1 positive area (%) in hippocampus or entorhinal (EC) /piriform cortex is shown. Unpaired two-tailed Welch’s t-test; *p < 0.05. *N* = 19–21 mice. Multiple sections per mouse were analyzed and averaged. (**C**) Human tau concentration in CSF of PS19 mice treated with or without TGN-073. Unpaired two-tailed Welch’s t-test; *p < 0.05. *N* = 4 mice. (**D**) Human tau concentration in RAB, RIPA, formic acid fractions of brain homogenates from PS19 mice with or without TGN-073. Unpaired two-tailed Welch’s t-test; *p < 0.05. *N* = 8–10 mice

Histological analysis with a phosphorylation-dependent anti-tau antibody PHF-1 revealed a significant reduction of tau pathology in both the cortex and hippocampus of TGN-073-treated PS19 mice, corresponding to approximately 37% reduction in the entorhinal/piriform cortex and 46% reduction in the hippocampus (Fig. 3A). Consistent results were obtained using a conformation-specific anti-tau antibody MC1 (Fig. 3B). In parallel, CSF analysis demonstrated a more than twofold increase in human tau in TGN-073–treated mice compared with controls (Fig. 3C), indicating enhanced glymphatic efflux of ISF-derived tau into the CSF.

Biochemical fractionation further demonstrated that insoluble tau species in formic acid extractable fractions were reduced by approximately 25%, whereas soluble tau levels in salt-containing reassembly buffer (RAB) or radio-immunoprecipitation assay buffer (RIPA) extractable fractions remained unchanged (Fig. 3D). Together, these results indicate that AQP4 activation facilitates clearance of extracellular tau species, thereby limiting their aggregation and propagation.

### AQP4 activation protects against neuronal loss in tauopathy

**Fig. 4:**
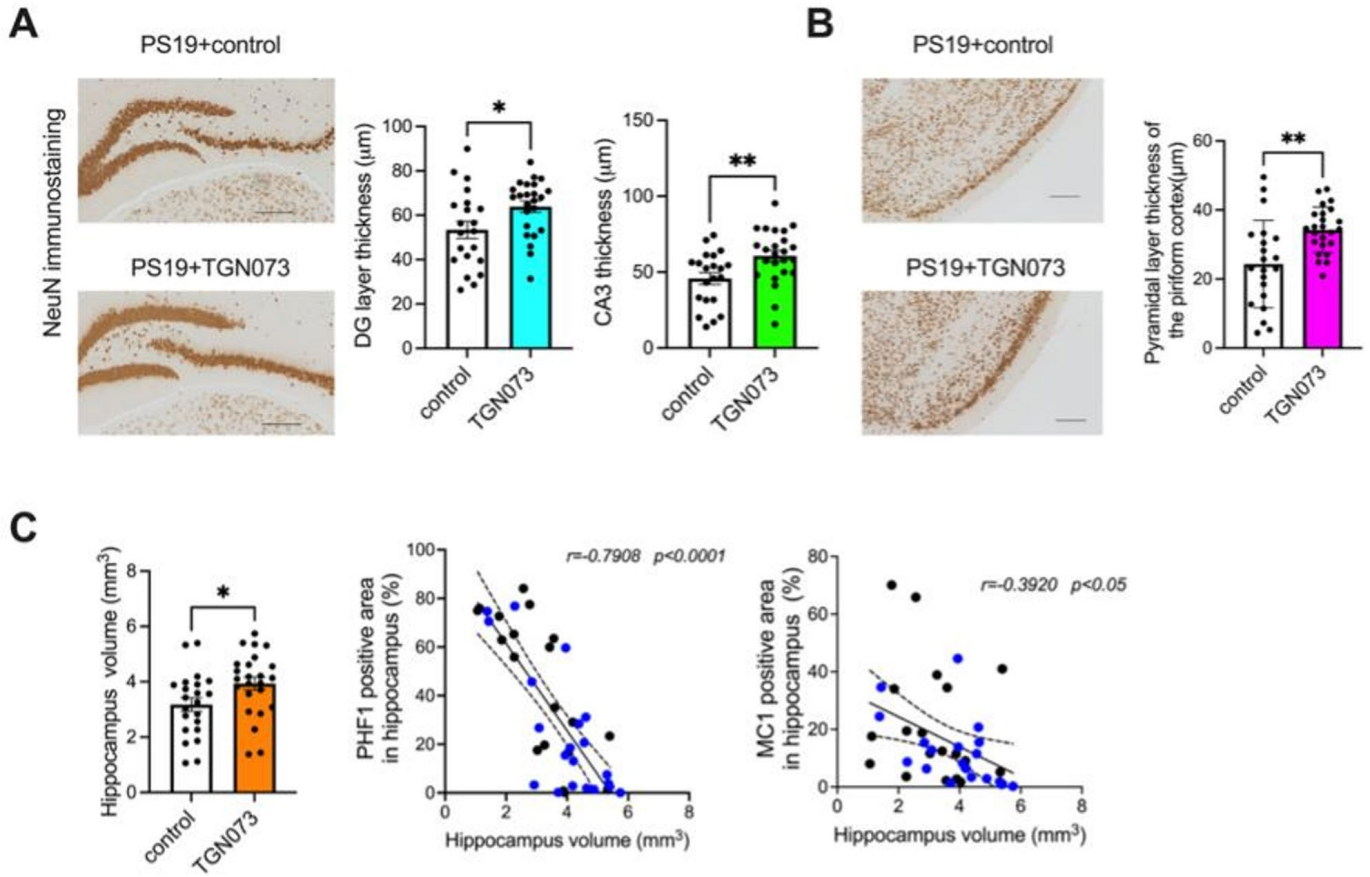
TGN-073 preserves neuronal structure and hippocampal integrity in PS19 mice. (**A**) Representative images of the hippocampus from 9-month-old PS19 mice treated with or without TGN-073 for 3 months, stained for NeuN. Scale bar, 200 μm. Quantification of dentate gyrus (DG) layer thickness and CA3 thickness is shown. Unpaired two-tailed Welch’s t-test; *p < 0.05, **p < 0.01. *N* = 21–24 mice. Multiple sections per mouse were analyzed and averaged. (**B**) Representative images of the piriform cortex from 9-month-old PS19 mice treated with or without TGN-073 for 3 months, stained for NeuN, along with quantification of the pyramidal cell layer thickness. Scale bar, 200 μm. Unpaired two-tailed Welch’s t-test; **p < 0.01. *N* = 21–24 mice. Multiple sections across rostrocaudal levels (approximately bregma − 2.0 to − 3.5 mm) were analyzed in defined regions of interest (hippocampus, piriform cortex, and entorhinal cortex) and averaged per mouse. (**C**) Quantification of hippocampal volume (hippocampal regions outlined by lines). Unpaired two-tailed Welch’s t-test; *p < 0.05. *N* = 21–24 mice. Multiple sections across rostrocaudal levels (approximately bregma − 2.0 to − 3.5 mm) were analyzed in defined regions of interest (hippocampus, piriform cortex, and entorhinal cortex) and averaged per mouse. Correlations between PHF-1– or MC1–positive area in the hippocampus and hippocampal volume are shown with linear regression curves and 95% confidence bands of the best-fit lines. Pearson r and p values are indicated. TGN treated mice were indicated as blue circles

To examine whether AQP4 activation confers neuroprotection, we quantified NeuN-positive neurons in vulnerable brain regions. TGN-073–treated PS19 mice exhibited significantly greater neuronal layer thickness in dentate gyrus (DG) (~ 20% increase) and the CA3 (~ 32% increase), as well as in the piriform cortex (~ 40% increase), compared with untreated controls (Fig. 4A, B). Morphometric analysis further revealed ~ 24% increase in hippocampal volume in TGN-073–treated PS19 mice (Fig. 4C).

Hippocampal volume correlated strongly and negatively with PHF-1-positive tau pathology (*r*=-0.78, *p* < 0.0001) and displayed a modest but significant correlation with MC1-positive misfolded tau (*r*=-0.39, *p* = 0.0124), indicating that reduced levels of tau accumulation—particularly those of aggregated or phosphorylated forms—is associated with amelioration of tau-related neuropathology (Fig. 4C). Thus, enhancing glymphatic function not only limits tau accumulation but also mitigates tau-induced neurodegeneration.

### Behavioral effects of chronic TGN-073 treatment in PS19 mice

Behavioral assessments were performed at 9 months of age following chronic TGN-073 treatment. Despite these robust histological improvements, TGN-073 did not significantly improve Y-maze performance (supplemental Fig. 1B). Limb clasping scores showed a mild trend toward improvement (supplemental Fig. 1C), while nest-building performance remained unchanged (supplemental Fig. 1D). These findings indicate that the histological benefits of TGN-073 were not accompanied by detectable behavioral improvement under the conditions tested.

### AQP4 activation attenuates glial activation in PS19 mice

**Fig. 5:**
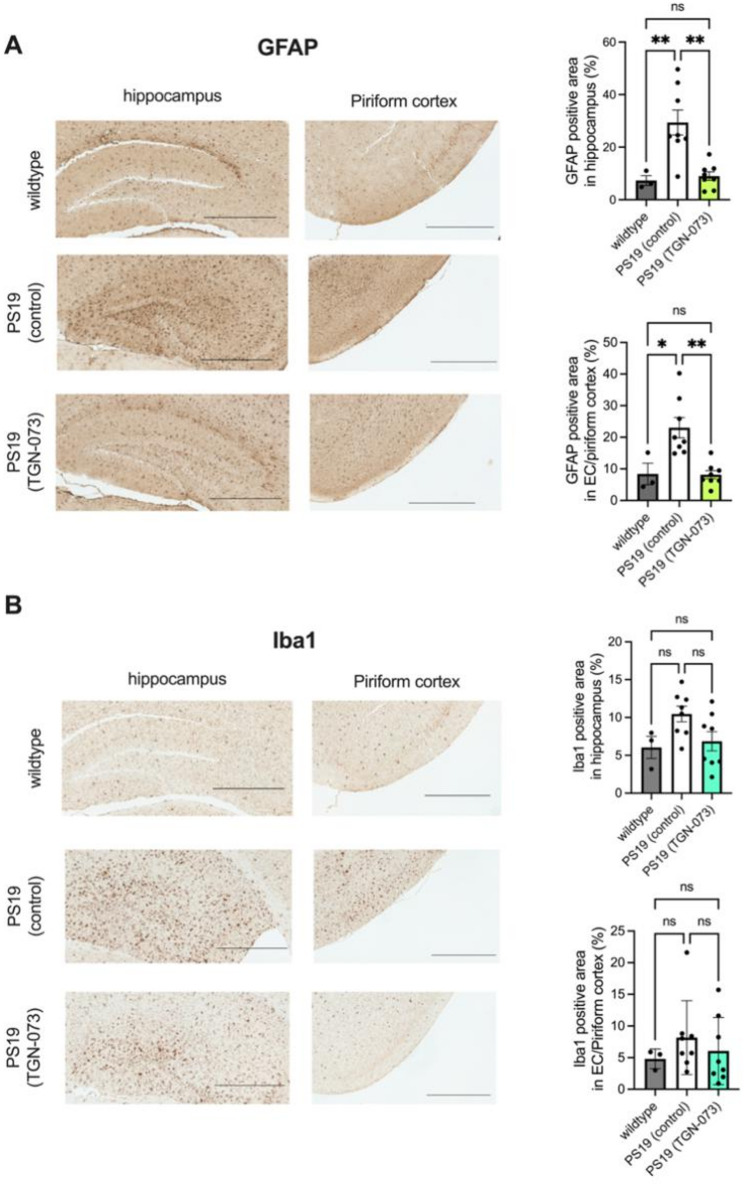
TGN-073 attenuates glial activation in PS19 mice. (**A**) Representative images of the hippocampus and piriform cortex from 9-month-old PS19 mice treated with or without TGN-073 for 3 months along with untreated wildtype mice, stained for GFAP. Scale bar, 500 μm. Quantification of GFAP positive area (%) in hippocampus or entorhinal (EC) /piriform cortex is shown. One-way ANOVA with Tukey’s post hoc test; *p < 0.05, **p < 0.01. *N* = 3–8 mice/group. Multiple sections per mouse were analyzed and averaged. (**B**) Representative images of the hippocampus from 9-month-old PS19 mice treated with or without TGN-073 for 3 months along with untreated wildtype mice, stained for Iba1. Scale bar, 500 μm. Quantification of Iba1 positive area (%) in hippocampus or entorhinal (EC) /piriform cortex is shown. One-way ANOVA with Tukey’s post hoc -test; *p < 0.05, **p < 0.01. *N* = 3–8 mice/group. Multiple sections per mouse were analyzed and averaged

Given the established contribution of neuroinflammation to tau-associated neurodegeneration, we next examined whether chronic TGN-073 treatment influences astrocytic and microglial activation in PS19 mice. Immunohistochemical analysis revealed marked GFAP upregulation in the hippocampus and cortex of PS19 mice compared with wild-type controls, which was significantly reduced following TGN-073 treatment (Fig. 5A). In contrast, changes in Iba1 immunoreactivity were more modest and region-dependent. Hippocampal Iba1 signals were increased in PS19 mice relative to wild-type controls and were reduced following TGN-073 treatment, although the overall three-group comparison did not reach statistical significance (Fig. 5B). In the cortex, Iba1 immunoreactivity showed little difference between wild-type and PS19 mice and only a trend toward reduction following treatment. These findings indicate that chronic TGN-073 treatment robustly attenuates astrocytic activation in PS19 mice, whereas its effects on microglial activation are comparatively modest.

### TGN-073 restores perivascular AQP4 enrichment without altering total AQP4 abundance

**Fig. 6:**
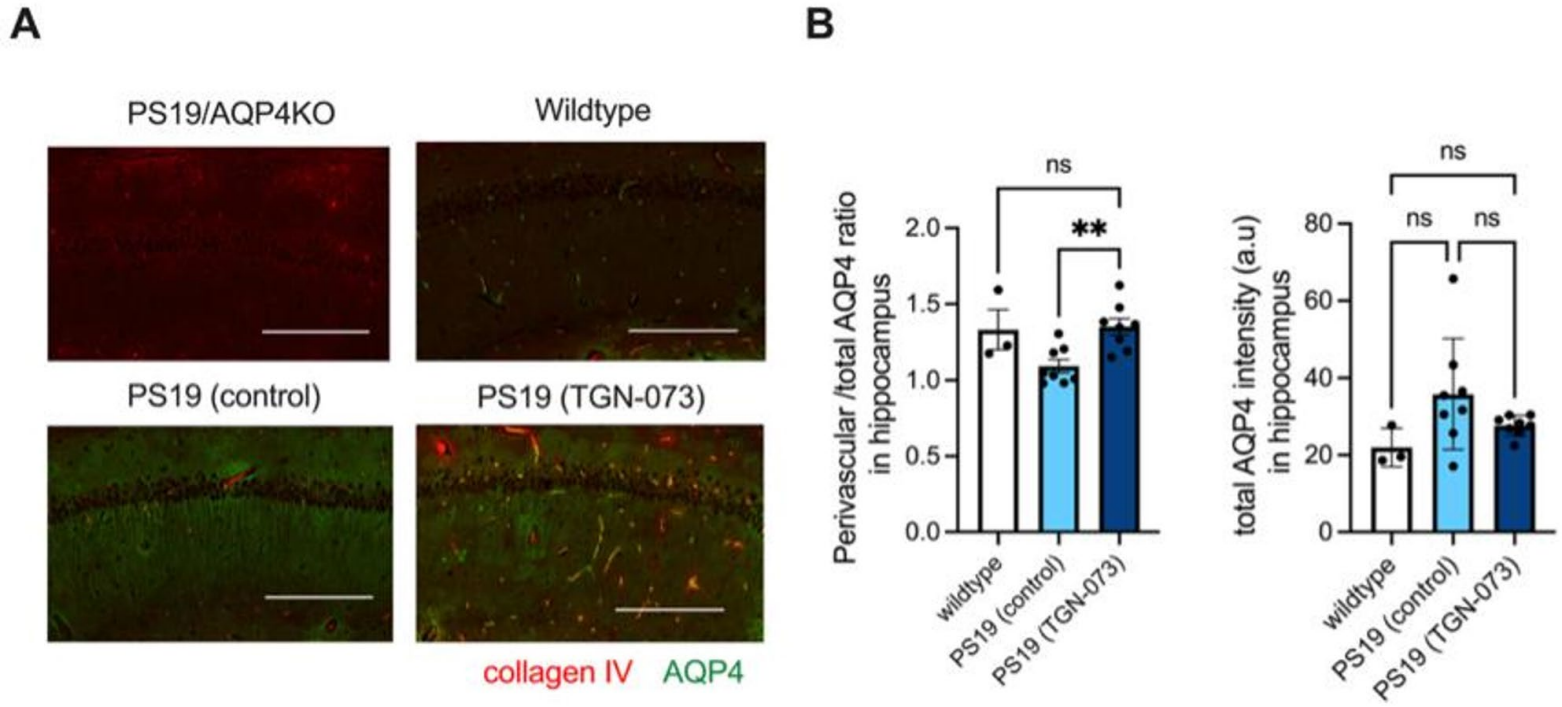
TGN-073 alters perivascular AQP4 in PS19 mice. (**A**) Representative images of the hippocampus from 9-month-old PS19 mice treated with or without TGN-073 for 3 months along with an untreated wildtype mouse and a PS19/AQP4KO mouse, stained for Collagen IV and AQP4. Scale bar, 200 μm. (**B**) Quantification of perivascular/total AQP4 in hippocampus as well as total AQP4 in hippocampus is shown. One-way ANOVA with Tukey’s post hoc-test; **p < 0.01. *N* = 3–8 mice/group. Multiple sections per mouse were analyzed and averaged

To investigate whether TGN-073 alters AQP4 expression or localization, we performed double immunofluorescence staining for AQP4 and collagen IV and quantified both total AQP4 fluorescence intensity and relative perivascular AQP4 enrichment. Relative perivascular AQP4 enrichment was significantly reduced in PS19 mice compared with wild-type mice, consistent with previous reports demonstrating impaired perivascular AQP4 localization in tauopathy models [15, 24]. Notably, TGN-073 treatment significantly increased perivascular AQP4 enrichment, restoring values to levels comparable to those observed in wild-type animals (Fig. 6A, B). In contrast, total hippocampal AQP4 fluorescence intensity showed a trend toward an increase in PS19 mice relative to wild-type controls but was not significantly altered by TGN-073 treatment (Fig. 6A, B). Similar findings were observed in the cortex (Supplementary Fig. 2). These results indicate that TGN-073 does not substantially alter overall AQP4 abundance but instead promotes perivascular enrichment of AQP4.

### The effects of TGN-073 are AQP4-dependent

**Fig. 7:**
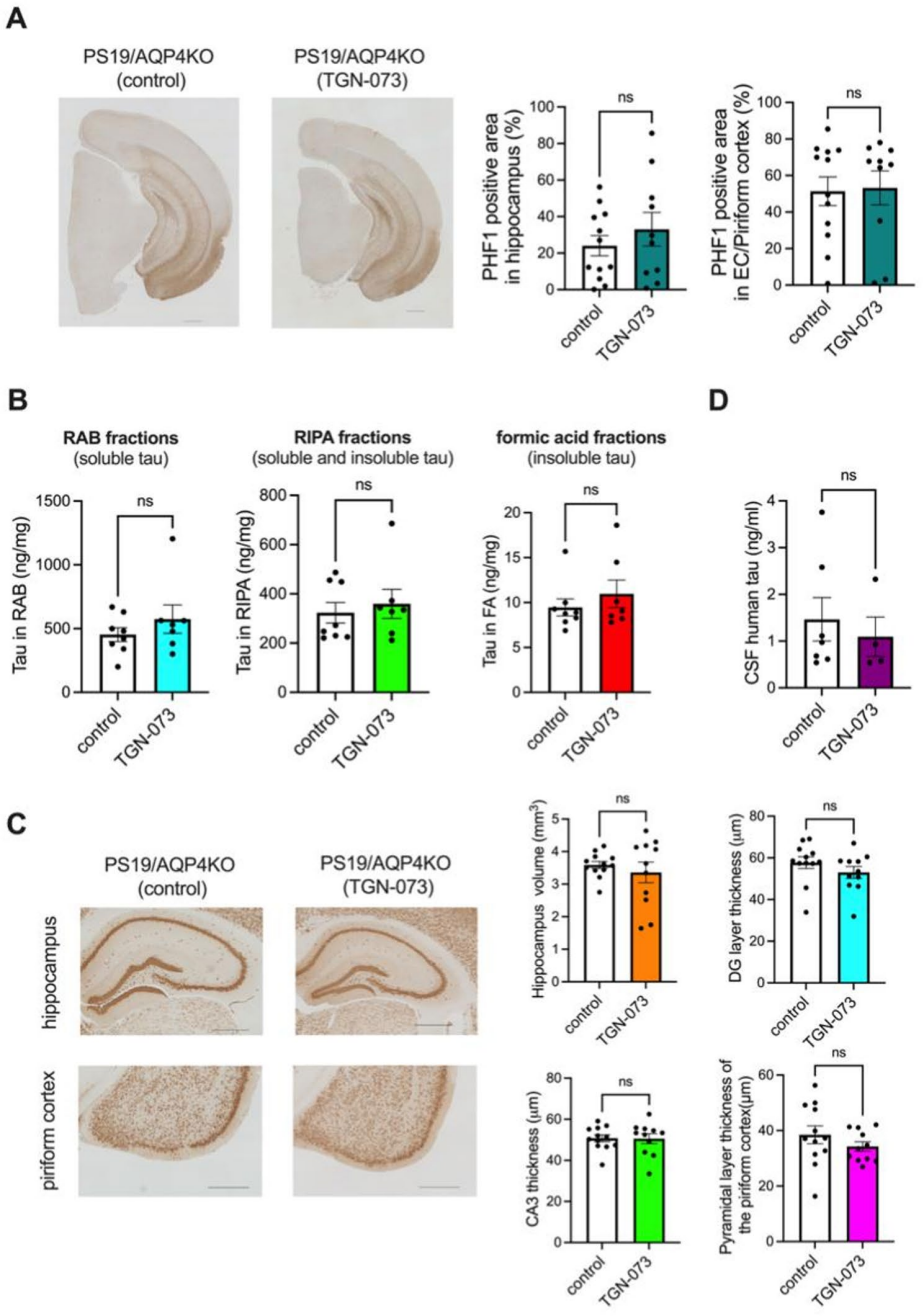
Loss of AQP4 abolishes the therapeutic effects of TGN-073 on tau pathology and neurodegeneration in PS19 mice. (**A**) Representative whole-brain images of 9-month-old PS19/AQP4KO mice treated with or without TGN-073, stained for PHF-1. Scale bar, 500 μm. Quantification of PHF-1 positive area (%) in hippocampus or entorhinal (EC) /piriform cortex is shown. Unpaired two-tailed Welch’s t-test. *N* = 10–12 mice. Multiple sections across rostrocaudal levels (approximately bregma − 2.0 to − 3.5 mm) were analyzed in defined regions of interest (hippocampus, piriform cortex, and entorhinal cortex) and averaged per mouse. (**B**) Quantification of human tau in RAB, RIPA and formic acid fractions of 9-month-old PS19/AQP4KO mice treated with or without TGN-073. Unpaired two-tailed Welch’s t-test. *N* = 7–8 mice. (**C**) Representative images of the hippocampus and piriform cortex from 9-month-old PS19/AQP4KO mice treated with or without TGN-073 for 3 months, stained for NeuN. Quantification of hippocampal volume, dentate gyrus (DG) layer thickness, CA3 thickness and the pyramidal cell layer thickness is shown. Scale bar, 500 μm. Unpaired two-tailed Welch’s t-test. *N* = 11–12 mice. Multiple sections per mouse were analyzed and averaged. (**D**) Human tau concentration in CSF of 9-month-old PS19/AQP4KO mice treated with or without TGN-073. Unpaired two-tailed Welch’s t-test. *N* = 4–7 mice. Note that the PS19 cohorts used in Figs. 3 and 7 were maintained on different genetic backgrounds; therefore, comparisons were performed within each experimental cohort

To determine whether the effects of TGN-073 are mediated by AQP4, we administered the compound to PS19 mice lacking AQP4 (PS19/AQP4 KO). In contrast to AQP4-intact PS19 mice, TGN-073 treatment did not reduce PHF-1- positive tau pathology (Fig. 7A), did not decrease biochemical tau burden across RAB, RIPA, and formic acid fractions (Fig. 7B), and did not ameliorate hippocampal volume loss or neuronal layer thinning (Fig. 7C). Moreover, whereas TGN-073 increased CSF human tau more than twofold in AQP4-intact PS19 mice, CSF tau concentrations were unchanged in TGN-073–treated PS19/AQP4 KO mice, indicating that AQP4 is required for TGN-073 to promote clearance of ISF-derived tau into the CSF (Fig. 7D).

We next examined whether the anti-inflammatory effects of TGN-073 also depend on AQP4. In contrast to AQP4-intact PS19 mice, TGN-073 treatment did not reduce GFAP or Iba1 immunoreactivity in PS19/AQP4 KO mice (supplemental Fig. 3A, B), indicating that the attenuation of glial activation by TGN-073 requires AQP4.

Together, these findings indicate that both the anti-tau, neuroprotective, and anti-inflammatory effects of TGN-073 require AQP4. Although complete dependence cannot be conclusively established, the absence of detectable benefit in AQP4-deficient mice strongly supports an AQP4-mediated mechanism.

## Discussion

This study demonstrates that pharmacological activation of AQP4 enhances glymphatic flow and is associated with reduced tau deposition and neuronal loss in PS19 tauopathy mice. Using JJ vicinal coupling proton exchange (JJVCPE) MRI with H_2_^17^O, we identified glymphatic impairment associated with tau and aging. Treatment with AQP4 activator TGN-073 increased glymphatic-related CSF–ISF tracer influx in acute DCE-MRI experiments, reduced pathological tau accumulation, and partially rescued neuronal loss in PS19 mice. These effects were largely absent in AQP4-deficient animals, supporting the conclusion that TGN-073 acts primarily through AQP4-dependent glymphatic enhancement.

Our findings build upon and mechanistically extend our previous report [13], which showed that AQP4 deficiency worsens tau pathology, by demonstrating that the level of AQP4 water-channel activity functionally influences tau accumulation and neuronal vulnerability. These data suggest that impaired glymphatic transport is not merely a downstream consequence of neurodegeneration, but an active contributor to disrupted protein homeostasis in tauopathy. Accordingly, the glymphatic pathway, and specifically AQP4-mediated water transport, represents a biologically modifiable mechanism that can influence disease progression.

Importantly, our histological analyses suggest that the effects of TGN-073 are not mediated by increased AQP4 expression. Although total AQP4 levels tended to be elevated in PS19 mice, perivascular AQP4 enrichment was reduced relative to wild-type mice. These findings suggest that tauopathy may disrupt the normal perivascular organization of AQP4 despite preserved or even increased overall AQP4 abundance. Notably, TGN-073 restored perivascular AQP4 enrichment to levels comparable to those observed in wild-type mice without significantly altering total AQP4 expression. Together with the loss of therapeutic efficacy in AQP4-deficient mice, these data suggest that modulation of AQP4 localization, rather than increased AQP4 abundance per se, may contribute to the enhancement of glymphatic function and reduction of tau pathology observed following TGN-073 treatment.

However, the present study does not establish the directionality of this relationship. Restoration of perivascular AQP4 may contribute to enhanced glymphatic-related transport, but it could also occur secondary to reduced tau pathology and gliosis. Further studies will be required to determine the causal relationship between AQP4 localization and disease progression.

In addition to reducing tau pathology, chronic TGN-073 treatment attenuated astrocytic activation and, to a lesser extent, microglial activation. Importantly, these effects were not observed in AQP4-deficient PS19 mice, indicating that suppression of gliosis is linked to AQP4-dependent mechanisms. Interestingly, astrocytic activation was normalized despite only partial reduction of tau pathology, raising the possibility that enhanced glymphatic transport may facilitate clearance of additional extracellular factors that contribute to neuroinflammatory responses. Future studies will be required to identify such mediators.

Using JJVCPE MRI, we identified a progressive impairment of glymphatic water exchange in PS19 mice that was evident at early disease stages, prior to overt insoluble tau deposition. This observation parallels clinical findings in primary tauopathies such as progressive supranuclear palsy, where reduced glymphatic function assessed by the DTI-ALPS index has been reported independently of amyloid pathology [28]. The early onset of glymphatic dysfunction suggests that soluble tau species, or early tau-associated neuronal or vascular alterations, may compromise fluid transport before the accumulation of fibrillar aggregates. Importantly, JJVCPE MRI enables quantitative assessment of water dynamics without reliance on exogenous tracers, providing a valuable approach for studying glymphatic alterations across disease stages in tauopathy models. It should be noted that JJVCPE MRI reflects water exchange dynamics and does not directly measure bulk CSF flow. Given differences in molecular size and transport mechanisms, water movement and CSF tracer flow are not equivalent. Therefore, our findings should be interpreted as alterations in glymphatic-related water exchange rather than direct measurements of CSF flow. Nevertheless, prior studies have shown that JJVCPE signals are sensitive to AQP4-dependent glymphatic function, and our combined pharmacological and genetic approaches further support the relevance of this measure.

To directly examine whether pharmacological modulation of AQP4 can enhance glymphatic transport in vivo, we employed dynamic contrast–enhanced MRI in wild-type mice and demonstrate that acute TGN-073 treatment increases glymphatic cerebrospinal fluid influx, particularly in cortical regions. These findings are consistent with prior reports in rats [29] and provide a mechanistic framework linking AQP4 activation to the reduction of tau pathology observed following chronic treatment in PS19 mice. Together, these data support the interpretation that sustained enhancement of CSF–ISF exchange contributes to limiting tau accumulation and neurodegeneration. The accompanying increase in cerebrospinal fluid tau levels is consistent with enhanced extracellular tau mobilization, supporting the notion that improved fluid exchange alters tau distribution dynamics. Although glymphatic function was not directly assessed by MRI following chronic TGN-073 treatment in PS19 mice, the AQP4-dependent reduction in tau pathology, attenuation of gliosis, increase in CSF tau levels, and neuroprotection collectively support sustained modulation of glymphatic-related processes.

Several physiological and behavioral factors—such as sleep [30], anesthesia [31], circadian rhythm [32], exercise [33] and neuronal activity [34, 35] —have been shown to enhance glymphatic flow, highlighting the importance of these pathways in maintaining brain homeostasis. These interventions, however, broadly influence multiple aspects of brain physiology, making it difficult to isolate the direct contribution of glymphatic enhancement. In contrast, selective pharmacological AQP4 activation provides a more direct approach, supporting a mechanistic contribution of glymphatic-related transport to the regulating tau accumulation.

Beyond tauopathy, our findings highlight a broader mechanistic principle linking brain fluid dynamics to intracellular proteostasis. Because impaired proteostasis and intracellular protein aggregation are shared features of multiple neurodegenerative diseases—including synucleinopathies, TDP-43 proteinopathies, and polyglutamine diseases—these results raise the possibility that targeting the glymphatic–proteostasis axis may have therapeutic potential beyond tauopathy. This conceptual framework integrates fluid dynamics with intracellular protein handling, revealing a mechanistic axis that has been largely underappreciated in the neurodegeneration field.

Several questions remain to be addressed. Although TGN-073 enhances glymphatic transport and suppresses tau pathology, the precise molecular mechanism by which AQP4 activation promotes the handling and removal of pathogenic tau species requires further investigation. Elucidating how enhanced water flux influences extracellular tau distribution, cellular uptake, and downstream aggregation will be critical for refining this therapeutic strategy. In addition, identification of the binding site and mode of action of TGN-073 on AQP4 would facilitate rational optimization of AQP4-targeting compounds. Although TGN-073 ameliorated tau pathology, neuronal loss, and gliosis, these histological benefits were not accompanied by significant behavioral improvement under the conditions tested. This may reflect the advanced stage of pathology at treatment onset or limited sensitivity of the behavioral assays. Future studies will be needed to determine whether glymphatic enhancement can translate into functional benefits.

In summary, our results provide proof-of-concept that pharmacological enhancement of glymphatic function using an AQP4 activator may mitigate tauopathy progression. By highlighting a potentially targetable CSF–ISF exchange–proteostasis axis associated with tau accumulation and neuronal vulnerability, this work offers a foundation for future development of glymphatic-based treatments for AD and related disorders. Furthermore, recent evidence showing that AQP4 activation also ameliorates amyloid pathology [36], suggests that modulation of brain fluid dynamics may offer a broadly effective therapeutic strategy targeting multiple pathological hallmarks of AD.

## Conclusions

This study demonstrates that glymphatic dysfunction emerges early and progressively worsens in a mouse model of tauopathy, and that pharmacological enhancement of AQP4-mediated water transport attenuates tau accumulation, gliosis and neurodegeneration. The loss of these protective effects in AQP4-deficient mice, together with altered CSF tau dynamics, supports a mechanistic link between AQP4-mediated glymphatic transport and tau proteostasis in vivo. These findings identify AQP4-dependent glymphatic transport as a potential disease-modifying target for tauopathies and related neurodegenerative disorders.

## Supplementary Information

Below is the link to the electronic supplementary material.

Supplementary Material 1

Supplementary Material 2

Supplementary Material 3

## Data Availability

The data that support the findings of this study are available from the corresponding author upon reasonable request.

## Abbreviations

CSF: Cerebrospinal fluid
AQP4: Aquaporin-4
AD: Alzheimer’s disease
