# Supplementary material for: AQP4-dependent enhancement of glymphatic function attenuates tau pathology and neurodegeneration in PS19 mice
Source: Mol Neurodegener. 2026 Jul 18;21:43. doi: 10.1186/s13024-026-00977-7 (PMC13450467; doi:10.1186/s13024-026-00977-7)
Supplement: Supplementary file 1 [file 13024_2026_977_MOESM1_ESM.pdf]

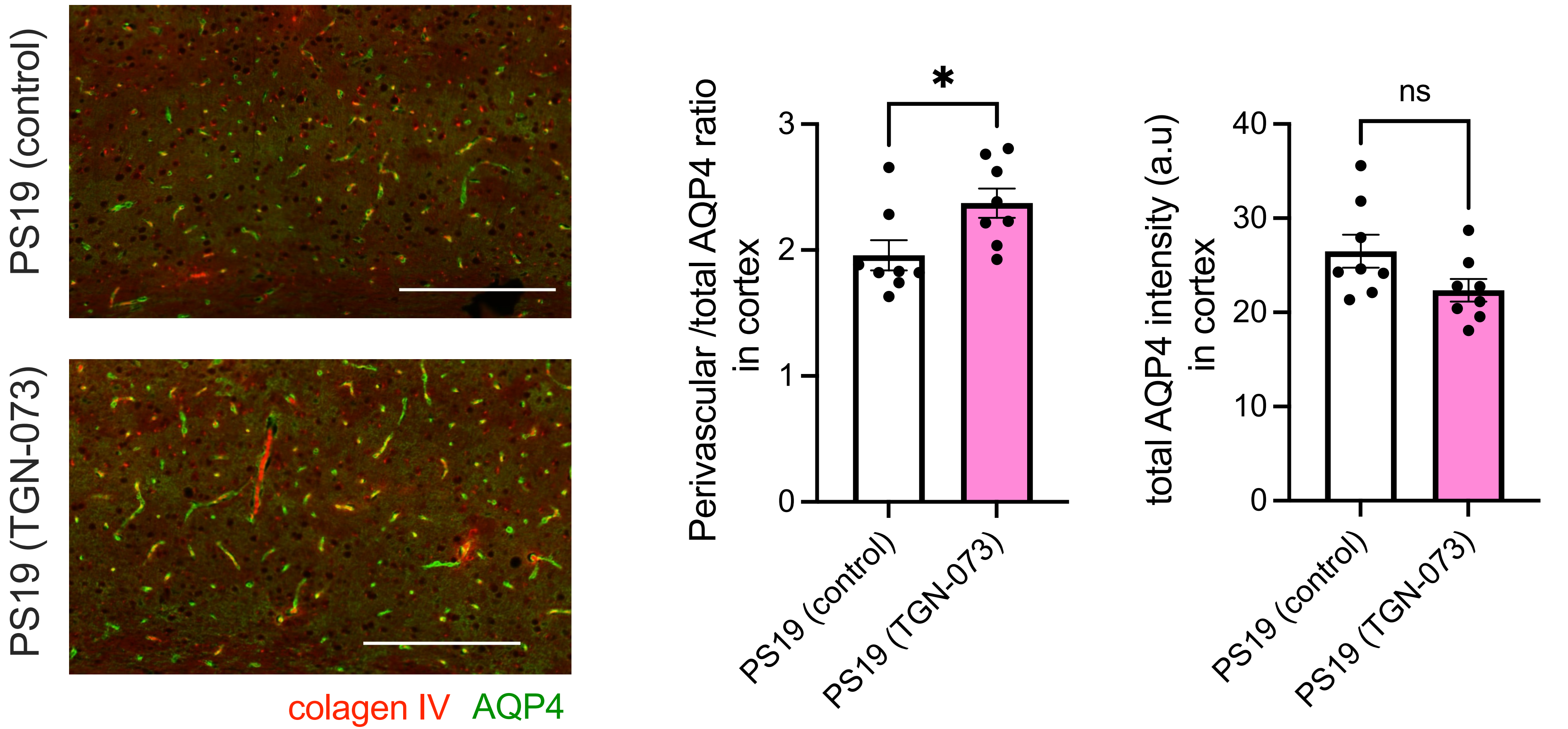

Supplemental Figure 2 The effect of TGN-073 on perivascular AQP4 in cortex of PS19 mice.
Representative images of the cortex from 9-month-old PS19 mice treated with or without TGN-073 for 3 months, stained for Collagen IV and AQP4. Scale bar, 200 mm. Quantification of perivascular/total AQP4 in the cortex as well as total AQP4 in hippocampus is shown. Unpaired two-tailed Welch's t-test. *p<0.05. N=8 mice/group. Multiple sections per mouse were analyzed and averaged.
