# Supplementary material for: AQP4-dependent enhancement of glymphatic function attenuates tau pathology and neurodegeneration in PS19 mice
Source: Mol Neurodegener. 2026 Jul 18;21:43. doi: 10.1186/s13024-026-00977-7 (PMC13450467; doi:10.1186/s13024-026-00977-7)
Supplement: Supplementary file 2 [file 13024_2026_977_MOESM2_ESM.pdf]

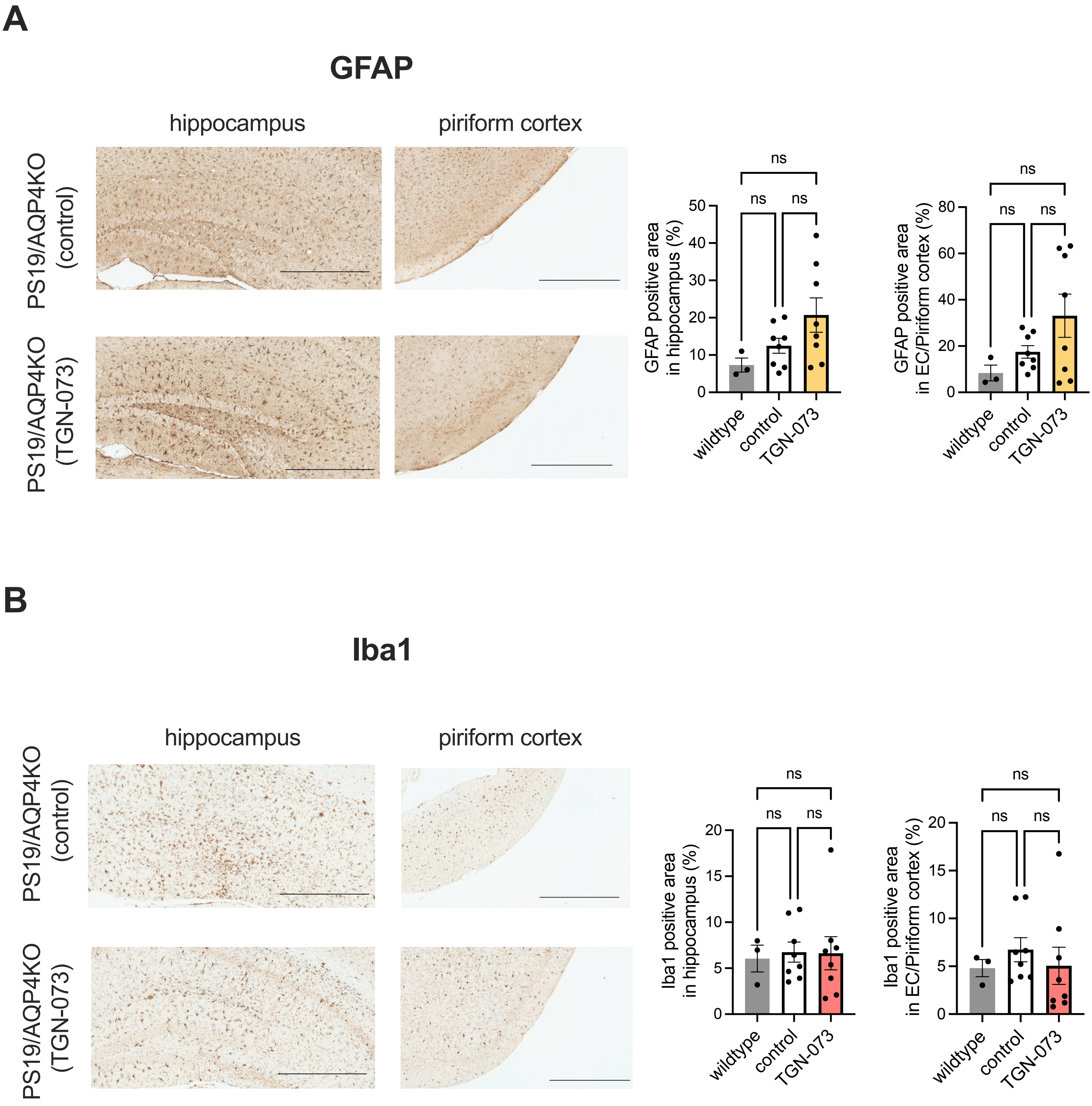

Supplemental Figure 3 Loss of AQP4 abolishes the therapeutic effects of TGN-073 on gliosis in PS19 mice. (A) Representative images of the hippocampus and piriform cortex from 9-month-old PS19/AQP4KO mice treated with or without TGN-073 for 3 months along with untreated wildtype mice, stained for GFAP. Scale bar, 500 mm. Quantification of GFAP positive area (%) in hippocampus or entorhinal (EC) /piriform cortex is shown. One-way ANOVA with Tukey's post hoc test. N=3-8 mice/group. Multiple sections per mouse were analyzed and averaged. (B) Representative images of the hippocampus from 9-month-old PS19/AQP4KO mice treated with or without TGN-073 for 3 months along with untreated wildtype mice, stained for Iba1. Scale bar, 500 mm. Quantification of Iba1 positive area (%) in hippocampus or entorhinal (EC) /piriform cortex is shown. One-way ANOVA with Tukey's post hoc-test. N=3-8 mice/group. Multiple sections per mouse were analyzed and averaged.
