# Supplementary material for: AQP4-dependent enhancement of glymphatic function attenuates tau pathology and neurodegeneration in PS19 mice
Source: Mol Neurodegener. 2026 Jul 18;21:43. doi: 10.1186/s13024-026-00977-7 (PMC13450467; doi:10.1186/s13024-026-00977-7)
Supplement: Supplementary file 3 [file 13024_2026_977_MOESM3_ESM.pdf]

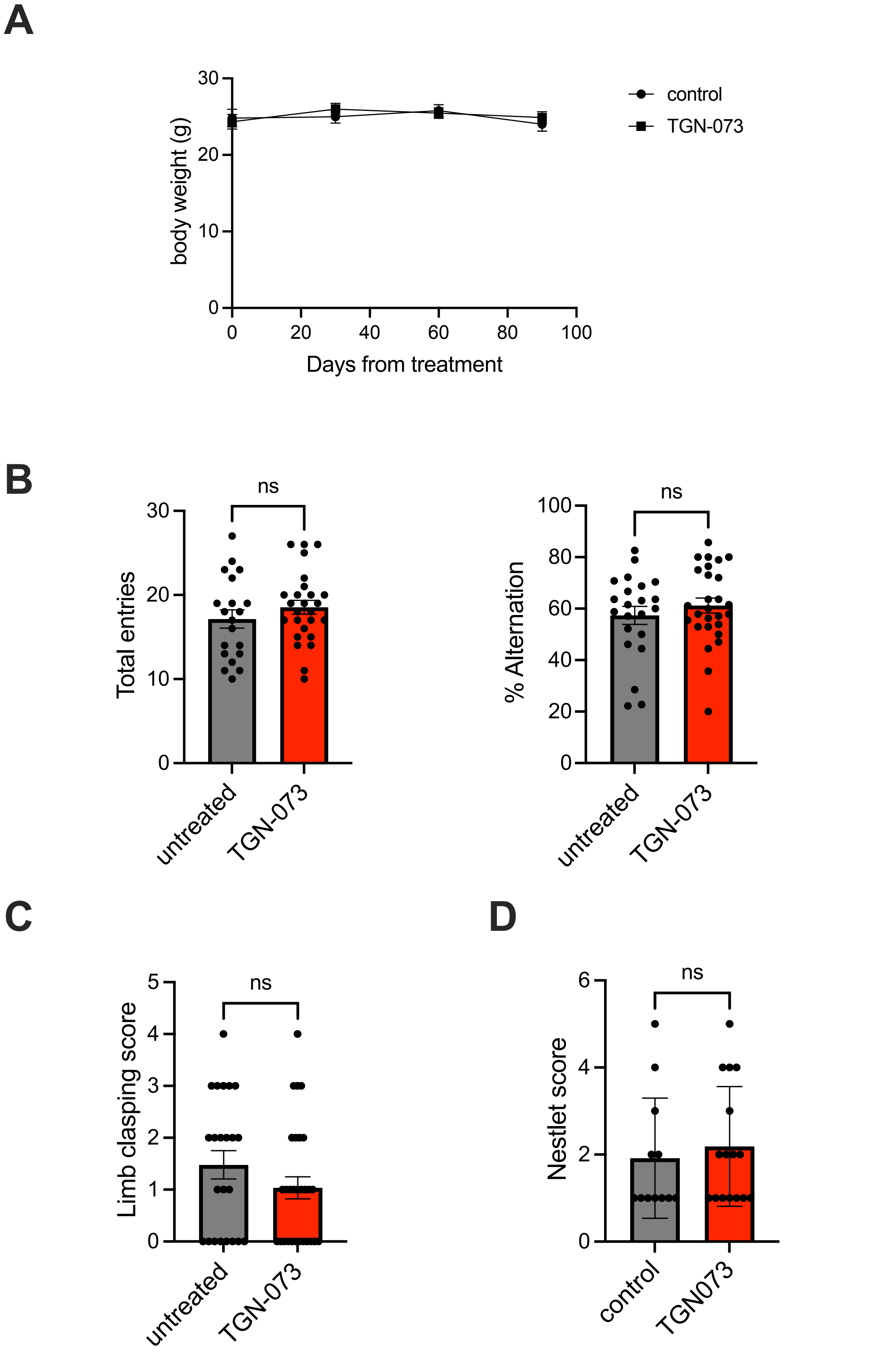

Supplemental Figure 1 Behavioral and physiological assessments during chronic TGN-073 treatment

Behavioral assessments were performed at 9 months of age following chronic TGN-073 treatment.

(A) Body weight changes in PS19 mice over the 3-month treatment period. N=17-21 mice. (B) Total arm entries and spontaneous alternation performance in the Y-maze spontaneous alternation test. Unpaired two-tailed Welch's t-test. N=20-26 mice. (C) Limb clasping scores. Mann-Whitney U test. N=23-29 mice. (D) Nestlet building performance. Mann-Whitney U test. N=12-16 mice.
